# Insulin Induces an Increase in Cytosolic Glucose Levels in 3T3-L1 Cells with Inhibited Glycogen Synthase Activation

**DOI:** 10.3390/ijms151017827

**Published:** 2014-10-02

**Authors:** Helena H. Chowdhury, Marko Kreft, Jørgen Jensen, Robert Zorec

**Affiliations:** 1 Celica, Biomedical, Tehnološki park 24, 1000 Ljubljana, Slovenia; E-Mails: helena.chowdhury@mf.uni-lj.si (H.H.C.); marko.kreft@mf.uni-lj.si (M.K.); 2 Laboratory for Neuroendocrinology–Molecular Cell Physiology, Institute of Pathophysiology, Faculty of Medicine, University of Ljubljana, Zaloška 4, 1000 Ljubljana, Slovenia; 3 Department of Biology, Biotechnical Faculty, University of Ljubljana, Večna pot 111, 1000 Ljubljana, Slovenia; 4 Department of Physical Performance, Norwegian School of Sports Sciences, P.O. Box N-4014 Ullevål Stadion, 0806 Oslo, Norway; E-Mail: jorgen.jensen@nih.no

**Keywords:** glucose, FRET, nanosensor, 3T3-L1 fibroblast, insulin, glycogen

## Abstract

Glucose is an important source of energy for mammalian cells and enters the cytosol via glucose transporters. It has been thought for a long time that glucose entering the cytosol is swiftly phosphorylated in most cell types; hence the levels of free glucose are very low, beyond the detection level. However, the introduction of new fluorescence resonance energy transfer-based glucose nanosensors has made it possible to measure intracellular glucose more accurately. Here, we used the fluorescent indicator protein (FLIPglu-600µ) to monitor cytosolic glucose dynamics in mouse 3T3-L1 cells in which glucose utilization for glycogen synthesis was inhibited. The results show that cells exhibit a low resting cytosolic glucose concentration. However, in cells with inhibited glycogen synthase activation, insulin induced a robust increase in cytosolic free glucose. The insulin-induced increase in cytosolic glucose in these cells is due to an imbalance between the glucose transported into the cytosol and the use of glucose in the cytosol. In untreated cells with sensitive glycogen synthase activation, insulin stimulation did not result in a change in the cytosolic glucose level. This is the first report of dynamic measurements of cytosolic glucose levels in cells devoid of the glycogen synthesis pathway.

## 1. Introduction

Glucose is a major source of metabolic energy for mammalian cells. For most cell types, glucose transport across the plasma membrane is directly coupled to metabolic pathways, such as glycogen, lactate, and fatty acid synthesis. The plasma membrane is impermeable to polar molecules such as glucose, therefore the cellular uptake of this important nutrient is accomplished by membrane-associated carrier proteins, glucose transporters (GLUT) that bind and transfer it across the lipid bilayer. GLUTs are expressed tissue specifically with different affinities for glucose [1].

In animals, one of the most important regulators of glucose homeostasis is insulin. This hormone is released from pancreatic beta cells after a meal and rapidly stimulates facilitative glucose transport mainly into skeletal muscle and adipocytes [2] by translocation of the insulin-sensitive glucose transporter GLUT4 to the plasma membrane [3]. Most other cell types express the constitutively active type of glucose transporter, GLUT1, which is to some degree also sensitive to insulin stimulation [4,5,6,7]. After glucose enters the cell, it is phosphorylated in the presence of hexokinase to glucose 6-phospate and is thus locked inside the cytosol. The metabolic fate of glucose in the cell may be oxidation for energy in the form of ATP and NADH; conversion to acetyl coenzyme A and subsequently to fatty acids; or it may be shunted into the pentose phosphate pathway to generate nicotinamide adenine dinucleotide phosphate (NADPH) and ribose 5-phosphate. Excess glucose is stored in the form of glycogen, which serves as secondary long-term energy storage. Glycogen synthase (GS) is a key enzyme in glycogenesis. The glycogen concentration is regulated by the complementary activities of glycogen phosphorylase (GP) and GS. GP catalyzes the degradation of glycogen to glucose 1-phosphate. GS is a rate-determining enzyme for glycogen synthesis with a hierarchal multisite phosphorylation mechanism [8].

From a systemic point of view, clearance of a high glucose level from the plasma is imperative as high plasma glucose concentrations lead to a variety of conditions, including insulin resistance, diabetes, metabolic syndrome, cardiovascular complications, *etc.* Therefore, understanding of glucose dynamics is of high importance and is the subject of study in many research groups. Several clinical studies deal with research on plasma glucose levels, thus it is also of particular interest to determine the intracellular dynamics of free glucose, especially in insulin-sensitive tissues.

It has long been suggested that glucose entering the cytosol is swiftly phosphorylated, rendering the concentration of free glucose beyond detection. However, this view has changed with the introduction of fluorescence resonance energy transfer (FRET)-based nanosensors, by which intracellular concentrations of ions and metabolites in living cells with high spatial and temporal resolution can be monitored. Hybrid proteins have been constructed and expressed in cells to measure the intracellular content of cyclic adenosine monophosphate (cAMP) [9,10]. Fehr *et al*. [11] used this approach to construct a FRET-based glucose nanosensor from a periplasmic glucose/galactose-binding protein with yellow and cyan fluorescent proteins attached, which has provided insights into glucose metabolism at the cellular level. With these sensors, glucose has been monitored in plant cells, COS-7 monkey kidney cells, mouse 3T3-L1 cells, astrocytes, and other cell types [12,13,14,15,16].

We recently reported cytosolic glucose measurements in 3T3-L1 adipocytes [13]. In the present study, we used 3T3-L1 cells expressing the FRET-based glucose nanosensor to monitor cytosolic free glucose concentrations in response to insulin treatment. Previous studies have shown that only a fraction of differentiated 3T3-L1 cells respond to insulin with increased cytosolic glucose [13], which is likely due to rapid utilization of free glucose in metabolic pathways. Therefore, we investigated whether insulin produced more robust increases in cytosolic free glucose in 3T3-L1 cells with insulin-resistant GS activation. 3T3-L1 cells were pretreated with serum-depleted medium to reduce the activity of GS [17]. The results show that the intracellular glucose concentration increases robustly in 3T3-L1 cells when GS is desensitized. This shows that insulin stimulates glucose transport into undifferentiated 3T3-L1 cells and that the metabolism of glucose depends on the ability to activate GS.

## 2. Results and Discussion

### 2.1. Dynamic Cytosolic Glucose Concentration in 3T3-L1 Cells

**Figure 1:**
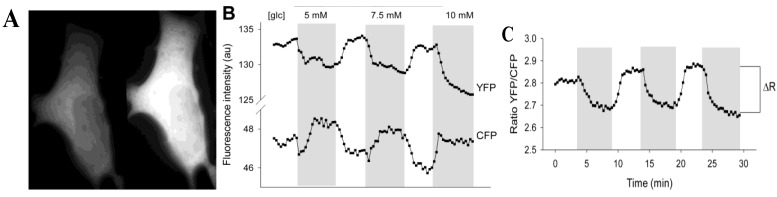
The extracellular glucose concentration modulates the fluorescence in 3T3-L1 cells expressing FLIPglu-600µ. (**A**) Photomicrographs showing fluorescence in a 3T3-L1 cell transiently expressing FLIPglu-600µ incubated without glucose. Using a dual view image splitter, the fluorescence of both fluorophores were separated and acquired simultaneously: cyan fluorescence (left panel, CFP) and yellow fluorescence (right panel, YFP); (**B**) Changes in the fluorescence intensity of YFP and CFP (recorded separately) in a single cell when incubated with different glucose concentrations (gray area) separated by 5-min incubations without glucose; (**C**) The YFP/CYP fluorescence intensity ratio was calculated from the data in (**B**). Δ*R* indicates the delta ratio, which is the difference between the average ratios obtained when the cells were incubated with and without glucose. The delta ratio is a measure of the change in the cytosolic glucose concentration.

3T3-L1 cells were transfected with the low-affinity glucose nanosensor, fluorescent indicator protein (FLIPglu-600µ), which was transiently expressed (Figure 1A). The percentage of 3T3-L1 cells expressing FLIPglu-600µ was around 1%. FLIPglu-600μ seemed to be equally distributed in the cytosol and was absent from the nucleus and some other organelles. To study changes in cytosolic free glucose with different concentrations of extracellular glucose supply, the yellow fluorescence/cyan fluorescence (YFP/CFP) ratio of the nanosensor was recorded. When external glucose was absent in the bathing solution, 3T3-L1 cells expressing FLIPglu-600µ showed relatively high yellow fluorescence. The addition of extracellular glucose rapidly decreased yellow fluorescence and increased cyan fluorescence (Figure 1B) due to accumulation of free glucose in the cytosol. When the extracellular glucose was removed, yellow fluorescence intensity reversibly increased and the YFP/CFP ratio returned to its initial level, indicating utilization and clearance of cytosolic glucose. Between the absence and the presence of high external glucose levels, the delta ratio (Δ*R*) was measured to determine the maximal change in cytosolic free glucose levels (Figure 1C). The slight steady decrease in the fluorescence intensity signal in both channels (Figure 1B) was likely due to probe bleaching. Figure 1C shows a representative recording of the average YFP/CFP ratio for an entire 3T3-L1 cell.

**Figure 2:**
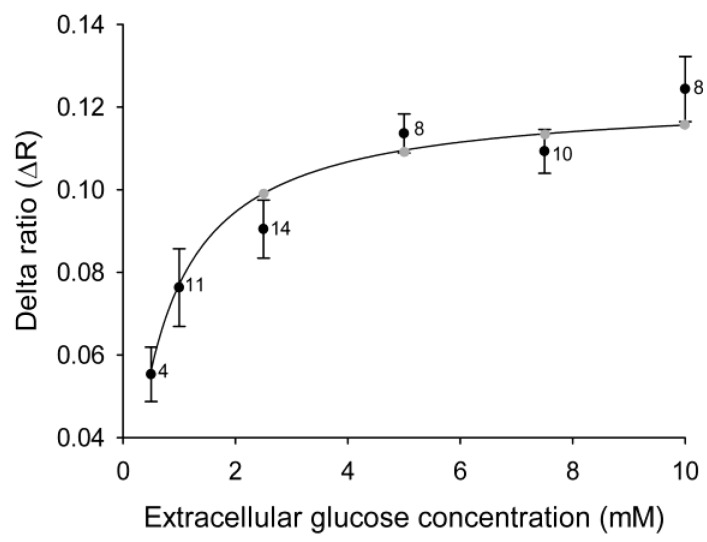
Cytosolic glucose levels in 3T3-L1 cells increase with increasing concentrations of extracellular glucose. Mean delta ratios (Δ*R*) reflect the cytosolic glucose levels in 3T3-L1 cells incubated with different concentrations of extracellular glucose. Each cell was incubated with 1–5 different glucose concentrations separated by 5-min wash periods without glucose, and the delta ratios were calculated as the difference between the average YFP/CFP ratios during incubation with and without glucose. The curve represents the best fit obtained by non-linear regression of the equation Δ*R* = (Δ*R*_max_ × [glc]_extra_)/(*K*_d_ + [glc]_extra_), with the gray symbols denoting the predicted Δ*R* at the extracellular glucose concentrations used in the experiments. The numbers adjacent to the symbols indicate the number of measurements of the explicit extracellular glucose concentration obtained from cells subjected to alternating extracellular glucose concentrations.

We bathed 3T3-L1 cells in increasing concentrations of external glucose with intermittent exposure to medium without glucose. Analysis of Δ*R* showed (Figure 1B,C) the effect of altering the glucose concentration gradient on the cytosolic glucose levels. A stepwise increment in the external glucose concentration increased the delta ratio in 3T3-L1 cells (Figure 2) until a steady state level was reached at around 5 mM extracellular glucose. The data on changes in the YFP/CFP ratio correspond well with previously measured changes in glucose in 3T3-L1 fibroblasts [13].

### 2.2. Insulin Induces an Increase in the Level of Cytosolic Glucose in 3T3-L1 Cells with Inhibited Glycogen Synthase Activation

**Figure 3:**
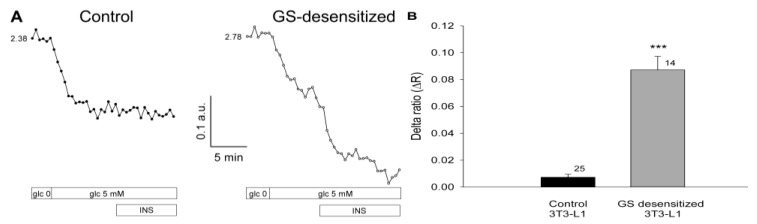
Insulin increases the intracellular glucose concentration in 3T3-L1 cells with reduced glycogen synthase (GS) activity. (**A**) Real-time fluorescence recordings in a control 3T3-L1 cell (**left**) and in a GS-desensitized 3T3-L1 cell (**right**). Desensitization of GS was achieved by preincubation of cells with 100 nM insulin. During imaging, the cells were first exposed to glucose-free medium, then to medium containing 5 mM glucose, with subsequent addition of 100 nM insulin. In both groups, control and GS desensitized, 5 mM extracellular glucose resulted in a decreased YFP/CFP ratio. In control cells, the addition of insulin had no effect on the YFP/CFP ratio, whereas in the GS-desensitized cells, a rapid and robust increase in the cytosolic glucose concentration was observed; (**B**) Mean delta ratio (YFP/CFP) after insulin stimulation in the control and GS-desensitized cells. The numbers adjacent to the bars indicate the number of measurements. Error bars show the standard error of the mean. Asterisks denote a significant difference (*** *p* << 0.001) compared with controls.

On entering the cell, glucose is metabolized through a variety of pathways, including the synthesis of glycogen. The key regulator enzyme in this metabolic pathway is GS, which is controlled by a variety of mechanisms [8,17]. Both glucose utilization and GS activation are regulated by insulin. To examine whether glucose levels in 3T3-L1 cells are dependent on the rate of glycogen synthesis under insulin-stimulated conditions, cells were subjected to a protocol that results in the specific desensitization of GS activation by insulin (see Materials and Methods). Cells were first inspected for fluorescence of the FLIPglu-600µ glucose nanosensor in glucose-free medium, and were then exposed to medium containing 5 mM glucose. Subsequently, insulin was applied as a bolus, reaching a final concentration of 100 nM. In both cell groups, control and GS-desensitized, a supply of 5 mM extracellular glucose resulted in a decreased YFP/CFP ratio (Figure 3A, left) indicating an increase in the intracellular glucose level, consistent with the results shown in Figure 2. In control cells, the addition of insulin had no effect on the YFP/CFP ratio (Figure 3A), likely due to regular activation of GS by insulin, which maintains a stable level of intracellular glucose. In contrast, in cells with desensitized GS activation, we observed a rapid and robust increase in cytosolic glucose concentration on stimulation with insulin. In GS-desensitized cells, insulin stimulation at a high glucose concentration resulted in a rapid and robust decrease in the YFP/CFP ratio (Figure 3A, right), indicating an increase in the cytosolic concentration of free glucose. The average results are summarized in Figure 3B. In control cells, insulin stimulation caused a small decrease in the YFP/CFP ratio, resulting in a ΔR of 0.007 ± 0.002. In GS-desensitized 3T3-L1 cells, insulin caused a significantly larger decrease in the YFP/CFP ratio, yielding a ΔR of 0.087 ± 0.010 (p << 0.001).

### 2.3. Discussion

The 3T3-L1 cell line, when differentiated into adipocytes, is a widely used cell model for studying insulin-sensitive metabolic and biochemical processes. The maturation of 3T3-L1 cells into adipocytes was studied in our previous work [13], where the results showed that differentiation of 3T3-L1 cells into adipocytes is associated with reduced glucose membrane permeability and changes in metabolism. We also addressed the responsiveness of the levels of cytosolic glucose to the application of insulin, which was found to be very low. Therefore, in this study we focused on 3T3-L1 undifferentiated cells, and we first examined the dynamics of intracellular glucose levels while exposing the cells to increasing concentrations of extracellular glucose. We used a FRET-based glucose nanosensor, FLIPglu-600µ. On binding of glucose to the nanosensor, a decrease in the fluorescence ratio between fluorophores (YFP/CFP) was observed, which was reversed on removal of glucose. A ratio change (*R*) after the addition of extracellular glucose is a measure of the increase in intracellular glucose (Figure 1), and the sensor was found to yield steady state levels at a concentration of approximately 5 mM extracellular glucose (Figure 2), consistent with a previous report [13], indicating that 3T3-L1 cells have constitutive plasma membrane glucose transport.

**Figure 4:**
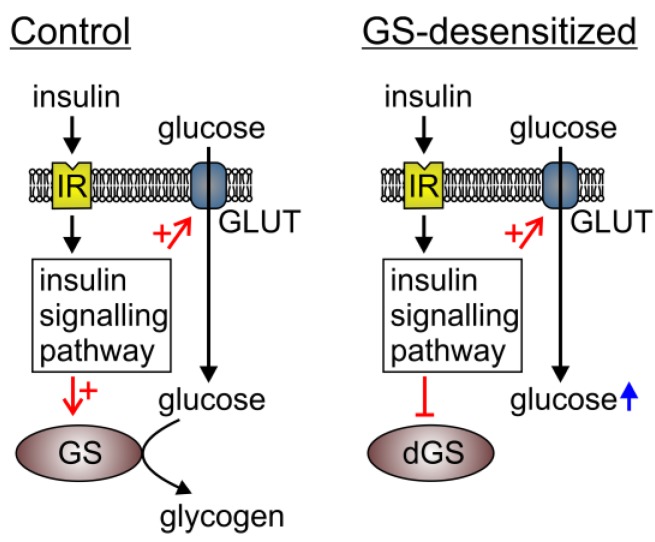
The mechanisms increasing the intracellular glucose level on insulin stimulation and GS desensitization in 3T3-L1 cells. In the basal state (Control), insulin stimulation does not result in increased intracellular free glucose, because glucose transport and its utilization are in equilibrium. In experiments where GS is resistant to insulin stimulation (dGS), insulin augments glucose transport, which exceeds the rate of glucose utilization. Hence, an increase in free glucose is measured. IR, insulin receptor; GLUT, glucose transporters; GS, glycogen synthase; dGS, desensitized glycogen synthase. The red arrow in combination with + indicates stimulation, red arrow with ┴ designates inhibition. Short black arrows indicate insulin signaling and long black arrows indicate glucose pathway. Blue arrow indicates increased glucose level.

We have investigated the cytosolic glucose levels in 3T3-L1 cells, which are insensitive to insulin-induced GS activation (GS-desensitized cells). In this way, one of the important metabolic pathways that consume glucose is truncated and it is believed that the glucose that enters the cytosol is not utilized by GS for glycogen synthesis (Figure 4) [17]. The results show that, in control cells, insulin has no significant effect on the cytosolic glucose concentration. This is expected, because expression of the insulin-sensitive glucose transporter, GLUT4, is not detected in 3T3-L1 cells [18]; this is also consistent with our previous study [13]. However, in the cells with desensitized GS [17], insulin stimulation resulted in a significant increase in cytosolic free glucose in the presence of 5 mM extracellular glucose. This is the first report of dynamic measurements of cytosolic glucose levels in cells devoid of the GS pathway. The cytosolic glucose concentration reflects the balance between glucose entry across the plasma membrane and cytosolic glucose utilization; therefore two processes stimulated by insulin could contribute to these results: increased glucose transport and/or increased/reduced glucose utilization. The latter would take place by the induction of a signaling pathway that results in the inhibition of hexokinase. It is generally accepted that the activity and abundance of the GLUT1 glucose transporter does not respond noticeably to insulin stimulation. It was shown, however, that not only GLUT4 but also GLUT1 is translocated to the plasma membrane in response to insulin [6]. Moreover, 3T3 MT3K fibroblasts, which express only GLUT1 glucose transporter, respond to insulin stimulation by increased glucose transport, and the counter control 3T3-HIR3.5, which expresses higher numbers of insulin receptors, responded to insulin with increased glucose transport as well as an increase in GLUT1 expression [5]. Furthermore, it was shown recently that in some cell lines and primary tumors, GLUT1 is the main glucose transporter, and in these cells insulin induces the translocation of GLUT1 to the plasma membrane, which is associated with increased glucose transport into the cell [4]. These reports demonstrate that GLUT1 may be insulin sensitive, albeit not to the same extent as GLUT4. The lack of change in glucose levels after insulin stimulation in control cells most probably displays a balanced rate of glucose influx and glucose cytosolic consumption, both stimulated by insulin. However, in cells with inhibited GS activation, at least some glucose utilization is eliminated; hence, excess glucose cannot be metabolized. This results in increased levels of glucose 6-phosphate, which in turn inhibits hexokinase and free glucose accumulates in the cytosol.

It has been demonstrated that in adipocytes pretreated with insulin, glucose transport is unaffected under basal and insulin-stimulated conditions [17]. Generally, glucose transport is considered to determine the rate of glucose metabolism in insulin-sensitive tissues. However, increased glycogen content reduces insulin-stimulated glycogen synthesis dramatically, whereas insulin-stimulated glucose uptake remains unchanged, indicating that glucose transport no longer determines the rate of glucose metabolism [19,20]. This is in line with our results, where we show increased glucose levels after insulin stimulation only in cells with inhibited GS activation. This process may be used in a new approach to glucose monitoring in patients with diabetes mellitus. Recently, a FRET-based glucose nanosensor was suggested for use in implantable glucose sensors for continuous glucose monitoring *in vivo* [21]. The sensor generates measurable FRET signals in response to glucose concentrations from 25 to 800 µM, which is suitable for measuring glucose noninvasively in body fluids such as tears and saliva [21]. Similarly, a nanosensor that senses a physiologic range of glucose plasma concentrations (0.05–11 mM) was used in the human liver carcinoma cell line, HEPG2 [22]. FRET-based glucose nanosensors have been used successfully in other cell types, such as plant [23] and yeast [24] cells, astrocytes, neurons, adipocytes, myoblasts, and tumor cells [14,15,16].

In conclusion, using the FRET-based glucose nanosensor, we show that in 3T3-L1 cells insensitive to insulin-stimulated GS activity, insulin augments the increase in the cytosolic glucose level, where glucose entry exceeds the reduced utilization of glucose for glycogen synthesis.

## 3. Experimental Section

### 3.1. Chemicals

Dulbecco’s modified Eagle’s medium (DMEM), fetal bovine serum (FBS), *N*-2-hydroxyethylpiperazine-*N*'-2-ethanesulfonic acid (HEPES), KCl, d-glucose (referred to as glucose throughout the article), NaH_2_PO_4_, NaHCO_3_, phosphate-buffered saline (PBS), trypsin and poly-l-lysine were purchased from Sigma*-*Aldrich (St. Louis, MO, USA). Sorbitol (d-glucitol) was obtained from Calbiochem (La Jolla, CA, USA). Insulin was purchased from Novo Nordisk (Bagsvaerd, Denmark). NaCl and NaOH were obtained from Merck (Darmstadt, Germany). CaCl_2_·6H_2_O was obtained from Riedel De Haen (Seelze-Hannover, Germany). MgCl_2_ was purchased from Kemika (Zagreb, Croatia), FuGENE (transfection reagent) was purchased from Roche (Basel, Switzerland).

### 3.2. Cell Culture and Cell Preparation

3T3-L1 cells were grown in DMEM containing 10% FBS, as described previously [18]. 3T3-L1 cells were seeded into tissue culture flasks (25 mL) and allowed to grow to 50%–80% confluence to maintain the fibroblast phenotype. Cells were cultured at 37 °C in 5% CO_2_. For experiments, cells were trypsinized and seeded onto coverslips coated with poly-l-lysine. Experiments were performed 2–6 days after cell preparation. Before the experiments, the cells were pretreated using the protocol described previously [17], which resulted in specific desensitization of GS activation by insulin. Briefly, cells were washed extensively with low serum medium (DMEM containing 5 mM glucose and l-glutamine) and were incubated in the same medium for 1.5 h. After incubation, the cells were stimulated with 100 nM insulin or vehicle only for 15 min, washed four times with PBS and incubated again in low serum medium for 1.5 h.

The bath solution for cell imaging consisted of (in mM): NaCl, 131.8; CaCl_2_, 1.8; KCl, 5; HEPES/NaOH, 10; NaH_2_PO_4_, 0.5; NaHCO_3_, 5 (pH 7.2). Glucose and sorbitol were added to this solution at various concentrations. Extracellular solutions with various glucose concentrations were added directly over the cells using a pipette.

### 3.3. Transfection

Plasmid DNA (FLIPglu-600µ) was extracted from *Escherichia coli* DH5α and purified using a commercially available protocol, the Pure Field Plasmid Midiprep system (Promega, Madison, WI, USA). Plasmid was introduced into the 3T3-L1 cells using the FuGENE transfection reagent. Briefly, cells were washed with serum-free medium and 100 µL of a mixture of serum-free medium, FuGENE and plasmid DNA was added to the cells and incubated for 60 min at 37 °C in 5% CO_2_. After transfection, serum was added to the cells and incubated in a humidified atmosphere at 37 °C in 5% CO_2_ for at least 24 h before the experiments.

### 3.4. Fluorescence Microscopy

Imaging was performed 24–48 h after nucleofection using a fluorescence microscope (Zeiss Axiovert 135; Zeiss, Oberkochen, Germany) equipped with a Till Photonics system and a CCD camera. Dual-emission intensity ratios were recorded using a monochromator (Polychrome IV; Till Photonics, Gräfeling, Germany) with 436 nm/10 nm excitation (neutral density filter with optical density = 0.6) and two emission filters (465 nm for CFP and 535 nm for YFP). YFP and CFP images were acquired simultaneously using a dual view image splitter (Optical Insights, Tucson, AZ, USA). For the analysis, the background fluorescence intensity was subtracted from the YFP and CFP emission. Images of fluorescent 3T3-L1 cells were acquired using a water-immersion objective (C-Apochromat, ×63, numerical aperture = 1.2) at intervals of 20 or 30 s. Exposure time was 700 ms.

### 3.5. Determination of Changes in the YFP/CFP Ratio and Statistical Analysis

The indicator protein, FLIPglu-600µ consists of fluorophores CFP and YFP fused to the *N* and *C* termini of glucose/galactose-binding protein. Glucose binding to glucose/galactose-binding protein causes a conformational change in the protein and consequently fluorophores move apart and FRET efficacy decreases. FRET was determined as the YFP/CFP emission intensity ratio of the background-corrected average fluorescence intensity over the entire cell. Changes in the ratio (Δ*R*) were calculated as the difference between average ratios during incubation with glucose-free medium and glucose medium.

Statistics are in the format of the mean ± the standard error of the mean. Differences between two samples were tested with the Student *t* test. Regression analysis was carried out by standard techniques (Sigma Plot software; SPSS, Inc., Chicago, IL, USA).

## 4. Conclusions

We describe the measurements on cytosolic glucose dynamics in 3T3-L1 cells using a fluorescent indicator protein, FLIPglu-600µ. In cells with inhibited GS activation, insulin induced a robust increase in cytosolic free glucose, whereas in cells with sensitive GS activation, insulin stimulation did not result in a change in the cytosolic level of glucose. The insulin-induced increase in cytosolic glucose in cells with inhibited GS activation is due to an imbalance between the amount of glucose transported into the cytosol and glucose utilization in the cytosol. This is the first report of dynamic measurements of cytosolic glucose levels in cells devoid of the GS pathway.
